# Exogenous Nitrogen Addition Reduced the Temperature Sensitivity of Microbial Respiration without Altering the Microbial Community Composition

**DOI:** 10.3389/fmicb.2017.02382

**Published:** 2017-12-01

**Authors:** Hui Wei, Xiaomei Chen, Jinhong He, Jiaen Zhang, Weijun Shen

**Affiliations:** ^1^Department of Ecology, College of Natural Resources and Environment, South China Agricultural University, Guangzhou, China; ^2^Key Laboratory of Agro-Environment in the Tropics, Ministry of Agriculture, Guangzhou, China; ^3^Guangdong Engineering Research Center for Modern Eco-Agriculture and Circular Agriculture, Guangzhou, China; ^4^School of Geographical Sciences, Guangzhou University, Guangzhou, China; ^5^Guangdong Provincial Key Laboratory of Applied Botany, South China Botanical Garden, Chinese Academy of Sciences, Guangzhou, China; ^6^College of Resources and Environment, University of Chinese Academy of Sciences, Beijing, China

**Keywords:** microbial communities, N deposition, greenhouse gas, SOM decomposition, *Q*_10_ index, global change

## Abstract

Atmospheric nitrogen (N) deposition is changing in both load quantity and chemical composition. The load effects have been studied extensively, whereas the composition effects remain poorly understood. We conducted a microcosm experiment to study how N chemistry affected the soil microbial community composition characterized by phospholipid fatty acids (PLFAs) and activity indicated by microbial CO_2_ release. Surface and subsurface soils collected from an old-growth subtropical forest were supplemented with three N-containing materials (ammonium, nitrate, and urea) at the current regional deposition load (50 kg ha^-1^ yr^-1^) and incubated at three temperatures (10, 20, and 30°C) to detect the interactive effects of N deposition and temperature. The results showed that the additions of N, regardless of form, did not alter the microbial PLFAs at any of the three temperatures. However, the addition of urea significantly stimulated soil CO_2_ release in the early incubation stage. Compared with the control, N addition consistently reduced the temperature dependency of microbial respiration, implying that N deposition could potentially weaken the positive feedback of the warming-stimulated soil CO_2_ release to the atmosphere. The consistent N effects for the surface and subsurface soils suggest that the effects of N on soil microbial communities may be independent of soil chemical contents and stoichiometry.

## Introduction

Atmospheric nitrogen (N) deposition has been recognized as an important aspect of global changes for decades, with various ecological and economic consequences observed around the world (Galloway et al., 2008; BassiriRad, 2015). N deposits are composed of oxidized/reduced and organic/inorganic N-containing materials and could change a series of ecosystem functions and processes, e.g., acidifying soils (Bobbink et al., 2010; Lu et al., 2015), reducing biodiversity (Bobbink et al., 2010; Lu et al., 2010; Clark et al., 2013), depressing soil respiration and CH_4_ oxidation but increasing N_2_O emissions (Mo et al., 2008; Zhang et al., 2008; Janssens et al., 2010), affecting litter decomposition (Mo et al., 2006; Hobbie, 2008; Chen et al., 2015), and increasing soil carbon (C) sequestration (Reay et al., 2008; Liu and Greaver, 2010; Chen et al., 2015). More importantly, N deposition could interact with other global change components, such as elevated CO_2_ concentration and warming (Hungate et al., 2009; Turner and Henry, 2009), and therefore make it difficult to clearly determine the ecological consequences by using single-factor experiments. With cascading effects on almost all ecosystem components, the effects of N deposition and the underlying mechanisms have been extensively studied in the past few decades (Galloway et al., 2008; BassiriRad, 2015).

In most of the previous studies, researchers have paid the most attention to the effects of N loading (Du et al., 2014; BassiriRad, 2015). However, changes in the chemical compositions of N deposits have been recorded in many places (Rogora et al., 2006; Liu et al., 2013; Hunova et al., 2017) possibly resulting from a combination of many factors, such as economic development policies, motor vehicles, the measures of reducing anthropogenic N emissions and the availability of reliable and cost-effective technologies (Erisman et al., 2001; Liu et al., 2013). The chemical composition of N deposits could vary over season or month in a calendar year (Fang et al., 2008). It is well known that different N-containing materials are involved in different biogeochemical processes (Corre and Lamersdorf, 2004; Du et al., 2014). However, whether change in the composition of N deposits result in diverse ecological consequences remains poorly understood.

Soil is the final receiver of atmospheric deposits, and underground ecological processes are vulnerable to atmospheric N deposition (Janssens et al., 2010; Cusack et al., 2011; Chen et al., 2015). Soil microbial communities play critical roles in these processes since they are the main drivers that are involved in almost all belowground processes (Balser et al., 2006). Previous studies have reported that soil microorganisms could respond rapidly to atmospheric N deposition (Allison et al., 2008; Cusack et al., 2011; Ramirez et al., 2012), and the responses could be ecosystem and N load specific (Treseder, 2008; Cusack et al., 2011; Zhou et al., 2012). The differences in N compositions among studies could partly contribute to these case-specific results because single N-containing chemicals are often used to simulate N deposition. The neglect of the effects of N composition may result in a biased understanding of the ecological consequences of N deposition.

The results of both meta-analyses and case studies suggest that N deposition suppresses soil microbial respiration (Janssens et al., 2010; Ramirez et al., 2010a), especially at highly productive sites with relatively less N limitations (Janssens et al., 2010). Such an effect may be attributed to two mechanisms: one mechanism involves enhanced chemical stabilization of soil organic C (SOC) by forming recalcitrant SOC that is highly resistant to microbial decomposition, and the other mechanism involves shifts in the substrate preference of soil microbes toward labile and energy-rich substrate (Janssens et al., 2010; Ramirez et al., 2012). Indirect effects of N additions (e.g., on soil pH) could not explain the consistent pattern of soil microbial respiration in response to N additions (Ramirez et al., 2010a). Besides, N form also affects the responsive magnitude of microbial respiration (Ramirez et al., 2010a). A recent study in a temperate forest showed that soil C cycling was inhibited by inorganic N, whereas it was promoted by organic N when both were applied at the same load (Du et al., 2014). These observations imply that the effects of N deposition could depend on deposited N chemistry. This dependence may result from different substrate preferences of various soil microbial groups (Treseder, 2008; Baran et al., 2015). For instance, ammonium can decrease fungal abundance, whereas nitrate mainly influences bacterial growth rates (Nunan et al., 2006; Birgander et al., 2014). Therefore, the altered composition of N deposits (e.g., the ratio of ammonium to nitrate) could alter soil microbial communities, although empirical evidence of this phenomenon remains scarce.

The microbial community composition and function in soil can be identified and evaluated by a variety of techniques (Torsvik and Øvreås, 2002; Leckie, 2005; Frostegård et al., 2011). Phospholipid fatty acid (PLFA) analysis is a widely used method for identifying community-level changes in microbial biomass and composition (Frostegård et al., 2011). Soil microbial respiration, i.e., CO_2_ emitted by soil microbial activities, is a synthesis index that reflects the function of the microbial community at decomposing soil organic matter (SOM) (Hanson et al., 2000; Janssens et al., 2010). In nature, soil microbial communities consist of thousands of microbial species that have different substrate preferences (Fierer et al., 2007; Baran et al., 2015). Diverse microbial groups respond differently to temperature increases, and these differences are accompanied by differing microbial community functions and temperature sensitivities (Wei et al., 2014). The thermal effects are sometimes due to limited substrate availability under warming conditions (Gershenson et al., 2009). As an important soil substrate, the N fertilization effects that are derived from atmospheric N deposition may alleviate such substrate limitations by promoting soil C and N availability, which could, therefore, confound the warming effects (Cusack et al., 2010; Chen et al., 2015).

By incubating surface and sub-surface soils collected from an N-rich subtropical forest (Fang et al., 2008), we conducted this microcosm experiment to understand the effects of N form and the interactions between N and temperature on soil microbial community composition and function. The two environmental factors, i.e., N and temperature, were studied, as atmospheric N deposition and warming have both been recorded for a long time (Erisman et al., 2001; IPCC, 2013; Liu et al., 2013). Both factors can significantly affect ecological processes and have received much attention across multiple ecosystems (Allison et al., 2010; Cusack et al., 2010; BassiriRad, 2015), and interactive effects likely exist between the two (Cusack et al., 2010; Chen et al., 2015; Liu et al., 2017). Microbial respiration could adapt to temperature and therefore have lower temperature dependence at high temperatures (Davidson and Janssens, 2006; Wei et al., 2014). Such a microbial acclimation may result from labile substrate depletion and microbial community adjustments in response to warming (Bradford et al., 2008; Allison et al., 2010; Wei et al., 2014). Atmospheric N deposition could alter soil substrate supply by alleviating soil C limitations and increasing inorganic N availability (Treseder, 2008; Chen et al., 2015), and alter soil microbial communities (Janssens et al., 2010; Ramirez et al., 2010b), consequently affecting microbial responses to temperature changes (Gershenson et al., 2009; Wei et al., 2014). However, inconsistent N and temperature interactions have been reported across N-rich forests (Mo et al., 2008; Cusack et al., 2010; Liu et al., 2017). Furthermore, the effects of N deposition on the temperature sensitivity of soil respiration may be greatly impacted by other factors such as stand age that can determine the direction of ecosystems in response to N addition (Mo et al., 2008; Deng et al., 2010). Great uncertainty still exists regarding the effects of N deposition across ecosystems, and explanation of the contradictions among studies is difficult, mainly due to the underlying mechanisms (including whether such effects depend on N composition) unclear.

In this study, we first aimed to examine the N-induced effects on soil microbial community PLFAs and SOM decomposition. We expected that the soil microbial community composition and function could respond rapidly to the experimental N addition and temperature manipulations. Furthermore, we expected that the microbial responses would depend on the applied N chemicals, because N species (e.g., ammonium vs. nitrate) can influence different microbial groups to different extents (Nunan et al., 2006; Birgander et al., 2014). Moreover, surface and subsurface soils with different physicochemical properties (see **Table 1**) were used to evaluate the treatment effects across soils. We expected that the surface soil would exhibit a greater response than the subsurface soil, as microbial responses to environmental changes depend greatly on soil properties and substrate availability (Karhu et al., 2010; Eberwein et al., 2015).

**Table 1:** Initial soil properties of the studied forest soils at the surface (0–10 cm) and subsurface (10–20 cm) soil layers.

	Surface soil	Subsurface soil	*p*
pH (KCl)	3.8 (0.1)	4.0 (0.1)	0.004
MBC (mg kg^-1^)	343.2 (13.6)	104.6 (20.9)	0.004
MBN (mg kg^-1^)	50.3 (6.9)	16.6 (7.4)	0.073
SOC (g kg^-1^)	40.2 (1.2)	15.3 (0.2)	<0.001
TN (g kg^-1^)	2.4 (0.1)	1.2 (0.0)	<0.001
TP (g kg^-1^)	0.23 (0.0)	0.19 (0.0)	0.036
C/N	16.6 (0.6)	13.0 (0.3)	0.002
N/P	10.4 (0.3)	6.1 (0.3)	0.006
C/P	173.2 (7.3)	79.5 (4.4)	0.002

*The numbers are the means with standard errors in the following brackets (*n* = 4) for the two soils, and the statistical *p-*values from the paired-sample *t-*tests are also presented. MBC stands for microbial biomass carbon, MBN for microbial biomass nitrogen, SOC for total soil organic carbon, TN for total nitrogen, and TP for total phosphorus. The C/N, N/P, and C/P stand for the C, N, P stoichiometry between two elements.*

## Materials and Methods

### Site Description

The soils were collected from two soil layers (0–10 and 10–20 cm) of an old-growth monsoon evergreen broadleaved forest, the climax vegetation in the Guangdong Province of southern China (112°30′ – 112°33′E, 23°09′ – 23°11′N). This region experiences a subtropical monsoon climate with the average annual air temperature of 22.3°C and the average precipitation of 1678 mm. The rainy season coincides with high temperature, with 80% of the annual precipitation occurring during the warm season from April to September. The studied forest is a mature forest with a stand age of approximately 400 years. It has naturally developed for hundreds of years under protection by the Buddhist monks in a neighboring temple (Tang et al., 2006). The dominant tree species in this forest include *Castanopsis chinensis, Schima superba, Cryptocarya chinensis, Machilus chinensis*, and *Syzygium rehderianum*. The soil in the studied forest is an acidic red soil that is similar to oxisols in USDA soil taxonomy (Tang et al., 2006).

### Experimental Design

Three factors, i.e., N form, incubation temperature and soil layer, were included in the present study. These factors included four N treatments [a control with no N addition and the addition of N in three forms (ammonium sulfate, potassium nitrate and urea)], three incubation temperatures (10, 20, and 30°C) and two soil depths (surface soil from 0 to 10 cm and subsurface soil from 10 to 20 cm). A full factorial design was employed in our study; thus, a total of 24 experimental treatments were conducted. For each treatment, four experimental replicates were used from four composite soil samples collected from the old-growth forest.

The three N chemicals were chosen because both inorganic (especially ammonium and nitrate) and organic N chemicals contribute to atmospheric N deposition in various ecosystems (Fang et al., 2008; Chen et al., 2015); however, few N deposition-related studies to date have considered organic N deposition (Cusack et al., 2011; Liu et al., 2013). For example, in the studied forest, organic N was found to contribute approximately 40% of the total N deposition load (18/50 kg N ha^-1^ yr^-1^) between 2004 and 2005 (Fang et al., 2008). The amount of N addition in the present study (3.79 mg N 50 g^-1^ dry soil) was approximately 50 kg N ha^-1^ for the 10 cm-depth soils, which is a value close to the N deposition load previously measured in this region (Fang et al., 2008).

The three incubation temperatures fall within the temperature range that occurs at the study site within a calendar year (Wei et al., 2015). The soil microbial respiration increased exponentially as the temperature increased within the range in the studied forest (Wei et al., 2015). These previous observations indicated that the soil microbial communities had not been exceedingly stressed by temperature in the present study, and the temperature sensitivity index *Q*_10_ could be calculated using the widely used exponential function as described in section “Data Analyses.” The two layers of soil were used to determine the treatment effects across soils, as the initial properties of the two soils were significantly different (see **Table 1**).

### Soil Collection and Incubation

Four quadrats with sizes of 15 m × 15 m and a buffering distance of at least 10 m between quadrats were established for collecting the soil samples. Within each quadrat, four 20 cm × 20 cm sampling spots were randomly located, and the visible plant materials were removed prior to soil sampling. The surface (0–10 cm) and subsurface (10–20 cm) soil samples that were collected from the four spots were composited into two samples – one surface soil sample and one subsurface soil sample. In total, four composite surface soil samples and four subsurface soil samples were taken as experimental replicates from the four quadrats. All samples were transported to the laboratory and passed through a 2-mm sieve to remove the visible stones and plant residues before the following analyses and incubation. Fresh soil samples were used to analyse the initial soil properties, and the results are shown in **Table 1**. As expected, the surface soil had lower soil pH but higher microbial biomass C (MBC) and N (MBN) and substrate content, including SOC, total N (TN), and total phosphorus (TP), compared to those of the subsurface soil. As a result, the C/N, N/P, and C/P ratios were significantly greater in the surface than the subsurface soil (*p* < 0.05, **Table 1**).

In this study, we had a total of 96 fresh soil samples (4 N forms × 3 temperatures × 2 soil layers × 4 replicates) for the incubation. Each sample, equivalent to 50 g of oven-dried soil weight, was placed into a 200 ml triangular glass flask. The soil water content of the incubation samples was adjusted to 50% of the water-holding capacity (WHC), after which the samples were incubated at room temperature (approximately 25°C) for 1 week to stabilize and restore the soil microbial communities. After stabilization, N solutions and deionized water were added to the soil samples (see Experimental Design for the N load determinations) to reach a final water content of 55% WHC, which falls within the optimal range of soil water content for the soil microbial activity in the studied forest soil (Zhou et al., 2014). The 96 soil samples were then incubated for 90 days in three thermostat incubators (RXZ-600B, Southeast Instrument Co., Ltd., Ningbo, China) set to the three temperatures, with each incubator containing 32 samples (4 N forms × 2 soil layers × 4 replicates).

During the incubation, a suitable amount of cotton was placed on the top of each incubation flask to prevent rapid water evaporation but permit gas exchange. Soil water content was checked by weighing the flasks twice per week, and deionized water was added to maintain the moisture level. On incubation days 1, 7, 12, 18, 26, 33, 42, 49, 56, 67, 80, and 90, two headspace gas samples were collected from each incubation flask using injection syringes. A three-way valve was installed on each syringe to maintain gas tightness before the determination of CO_2_ concentrations. The time interval between the two gas collections was 30 min per flask. To determine the headspace CO_2_ concentrations, following their collection, these gas samples were immediately analyzed in a gas chromatograph (Agilent 7890A, Agilent Technologies Inc., CA, United States) with a flame ionization detector (FID). The rate of CO_2_ emission was estimated as the slope of the CO_2_ concentration increase with time. At the end of the incubation, soil samples were collected for PLFA analysis.

### Soil Chemical and PLFA Analyses

The pH of soil suspensions at a ratio of soil to KCl solution of 1:2.5 was tested. The soil MBC and MBN were analyzed using the chloroform and fumigation method (Vance et al., 1987), and the C and N contents in the extracts were determined using a C analyzer (TOC-VCSH, Shimadzu Corp., Kyoto, Japan); the extraction coefficients used were 0.45 for the MBC and 0.54 for the MBN calculations (Brookes et al., 1985; Wu et al., 1990). The SOC was analyzed using the Walkley–Black method, while TN and TP were analyzed using the acid-digestion and colorimetric methods, respectively (Liu, 1996).

The PLFA analysis was conducted following Bossio and Scow (1998). Briefly, 5 g of freeze-dried soils were extracted by a buffering solution with a volume ratio of 1:2:0.8 of chloroform, methanol and citrate for each sample. The phospholipids in the organic phase were then separated and collected using a silica column (500 mg, ANPEL Laboratory Technologies Inc., Shanghai, China). After the samples were methanolysed by adding a mildly alkaline solution, the fatty acids were identified on an Agilent 7890 GC equipped with a Sherlock Microbial Identification System (Version 6.2, MIDI Inc., Newark, DE, United States). The individual PLFA contents (nmol g^-1^ soil) were determined based on the added internal standard (19:0), and the relative abundance of microbial groups (mol%) was calculated. Gram-positive (G+) bacteria were indicated by the sum of iso- and anteiso-fatty acids, while Gram-negative (G-) bacteria were indicated by the sum of cyclopropane fatty acids, mono-unsaturated fatty acids and hydroxyl fatty acids. Carboxylic acids with methyl functioning on the tenth C were regarded as actinomycetal indicators, and fungi were indicated by the summed abundance of 18:2w6c and 18:1w9c. Finally, the ratios of fungal to bacterial PLFAs and G+ to G- bacterial PLFAs were calculated to indicate the soil microbial community structure.

### Data Analyses

The accumulated soil CO_2_ emission (*C*_m_) values were calculated using an integrating method by multiplying the instantaneous CO_2_ emission rate by the duration of the incubation (Martin Wetterstedt et al., 2009). The entire incubation period was separated into three stages with stage 1 being the first 7 days, stage 2 being days 8–26 and stage 3 being the remaining period, and the accumulated CO_2_ release within the three stages was calculated. In each stage, the temperature sensitivity, presented as the *Q*_10_ value that indicates the *C*_m_ increase for every 10°C temperature increase (Arevalo et al., 2012), was also calculated following the commonly used *Q*_10_ function (Zhou et al., 2014):

$$\begin{array}{c} Q_{10}=e^{10b} \end{array}$$

The parameter b was obtained by fitting *C*_m_ with the incubation temperature (T) using the first-order exponential function (Arevalo et al., 2012):

$$\begin{array}{c} C_{m}=a\times e^{\mathrm{bT}} \end{array}$$

Although *Q*_10_ can vary with temperature (Lloyd and Taylor, 1994), the apparent temperature sensitivity remains useful for determining implications for soil CO_2_ emissions in response to temperature changes under existing environmental constraints (Davidson and Janssens, 2006).

Paired-sample *t*-tests were employed to detect significant differences in the initial properties between the surface and sub-surface soils. A three-way analysis of variances (ANOVA) was conducted on the base-e logarithms of each of the soil CO_2_ emissions in the three stages, and a repeated measures ANOVA was used to explore the significance of *Q*_10_ among N treatments over time. In each stage, one-way ANOVA with Tukey multiple comparisons was further conducted to compare the means among the treatments when normality and homoscedasticity hypotheses were met; otherwise, a Welch test with the Games-Howell method was used. Principal component analysis (PCA) was employed on the PLFA dataset to extract the principal components that were used for the further analyses. The scores of the first two PCs were correlated with total PLFAs, the relative abundances of the different microbial groups and the ratios of G+/G- and F/B to detect which microbial community properties contributed to the observed treatment effects on soil microbial community composition. The significance level was set at *p* < 0.05. All statistical analyses were finished in IBM SPSS statistics (version 22, IBM Corp., New York, NY, United States), except for *Q*_10_, which was estimated in R software (version 3.1.2).

## Results

### Effects on Soil CO_2_ Emission

**FIGURE 1:**
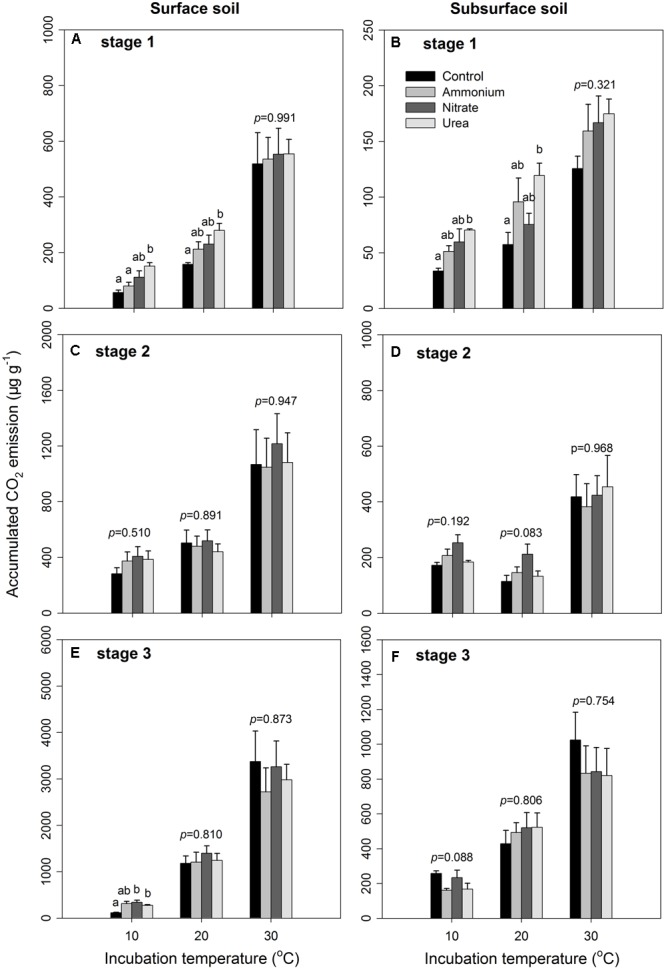
Accumulated CO_2_ emissions under different incubation temperatures and N additions for the surface and subsurface soils. The left three panels **(A,C,E)** represent the three incubation stages (0–7, 8–26, and 27–90 days) for the surface soil, respectively, while the right three panels **(B,D,F)** represent the three stages for the subsurface soil. The bars represent the means, and the error bars are the standard errors (*n* = 4). In each panel, the different lowercase letters above the bars indicate significant differences at *p* < 0.05 among the N treatments under each of the incubation temperatures. The statistical *p*-values were given when *p* > 0.05.

The results of the three-way ANOVA showed that the different N forms did not exhibit significantly different effects on the soil CO_2_ emissions as expected, except in stage 1 (*p* < 0.001, **Table 2**). The incubation temperature and soil layer significantly affected the *C*_m_ in the three stages (*p* < 0.001 for all, **Table 2**). N form did not show any significant interactive effects in conjunction with incubation temperature (*p* > 0.05 in all the three stages, **Table 2**) or soil layer in the first two stages (*p* > 0.05, **Table 2**). However, incubation temperature exhibited significant interactive effects with soil layer in the three stages (*p* < 0.001, **Table 2**). The interactions among the three factors (i.e., N form, temperature and soil layer) were only significant in stage 3 (*p* = 0.004, **Table 2**). In particular, the accumulated CO_2_ release was significantly higher under urea addition than that under the control at the two lower temperatures (10 and 20°C) in stage 1 (*p* < 0.05, **Figures 1A,B**), which was associated with the highest CO_2_ release at the beginning due to the addition of urea (Supplementary Figures S1, S2) as well as at 10°C during stage 3. N addition with either of the other N forms did not significantly affect the CO_2_ release (*p* > 0.05, **Figure 1** and **Table 2**), with the exception of nitrate addition at 10°C in stage 3. During the entire period, nitrate addition tended to cause relatively higher CO_2_ emissions than did ammonium addition, but the differences were not statistically significant (**Figure 1** and Supplementary Figures S1, S2). The surface soil released significantly more CO_2_ than did the subsurface soil under all the combinations of incubation temperature and N form (*p* < 0.05, **Figure 1** and **Table 2**), and the soil CO_2_ emissions significantly increased as the temperature increased (*p* < 0.001, **Figure 1** and **Table 2**).

**Table 2:** Summary of three-way ANOVAs on soil CO_2_ release at the three stages.

Source	Stage 1		Stage 2		Stage 3	
	*F*	*p*	*F*	*p*	*F*	*p*
N form	17.80	<0.001	2.58	0.060	1.67	0.182
T	201.22	<0.001	77.58	<0.001	366.34	<0.001
Soil layer	254.25	<0.001	174.56	<0.001	172.77	<0.001
N form ^∗^ T	2.10	0.064	0.45	0.844	1.47	0.201
N form ^∗^ Soil layer	0.34	0.797	0.25	0.864	3.34	0.024
T ^∗^ Soil layer	10.84	<0.001	7.77	0.001	26.91	<0.001
N form ^∗^ T ^∗^ Soil layer	0.45	0.843	0.53	0.785	3.54	0.004
*R*^2^/adjusted *R*^2^	0.91/0.88		0.83/0.78		0.93/0.91	

*The three independent factors are nitrogen (N) form, incubation temperature (T), and soil layer. Statistical *F-, p-*values and *R*^2^/adjusted *R*^2^ are presented in the table.*

**FIGURE 2:**
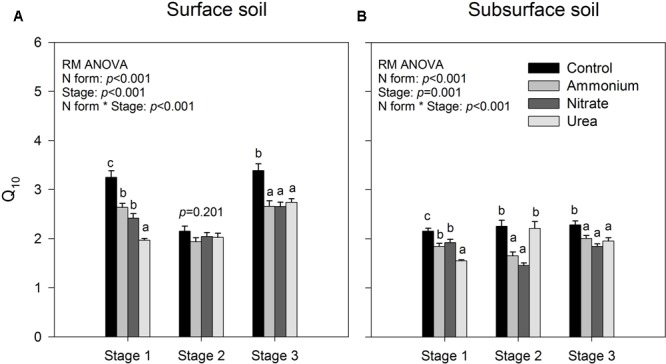
Temperature sensitivity (*Q*_10_) of accumulated CO_2_ release for the surface **(A)** and subsurface **(B)** soils. Stages 1–3 correspond to days 0–7, 8–26, and 27–90, respectively. The bars represent the means, and the error bars are the standard errors (*n* = 64). In each panel, the different lowercase letters above the bars indicate significant differences at *p* < 0.05 among the N treatments at each of the three stages. The statistical *p*-values were given when *p* > 0.05.

The results of repeated measures ANOVA showed that N form and incubation duration had significant effects on the temperature index *Q*_10_ for both of the surface and sub-surface soils, and their interactions were also significant (*p* < 0.001, **Figure 2**). Relative to the control, exogenous N addition decreased the temperature sensitivity of soil CO_2_ emissions (*p* < 0.05, **Figure 2**), except that decrease was not significant in stage 2 for the surface soil and for the subsurface soil with urea addition (*p* > 0.05, **Figure 2**). Among the three N forms, urea addition induced significantly lower *Q*_10_ than did the addition of the other two forms in stage 1 (*p* < 0.05, **Figure 2**), while there was no significant difference between ammonium and nitrate addition throughout the investigation period (*p* > 0.05, **Figure 2**). However, interactions between N form and temperature were not significant on the specific respiration (*C*_m_ values divided by total PLFA amount), indicating that *Q*_10_ was not significantly different among N treatments (*p* > 0.05, Supplementary Table S1). Except for the control and urea treatment in stage 2, the surface soil had significantly greater *Q*_10_ values than the subsurface soil (*p* < 0.05, **Figure 2**).

### Effects on Soil Microbial Community Composition

**FIGURE 3:**
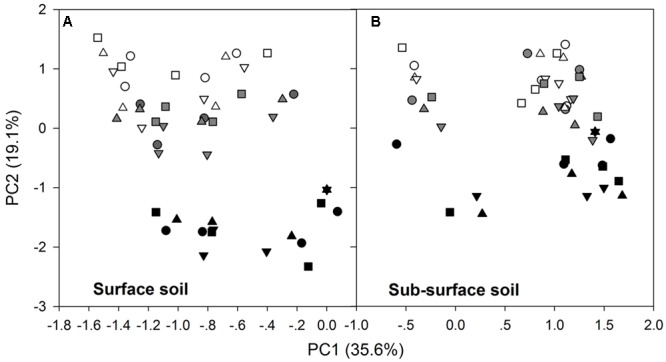
Scores of the first two principal components for the surface **(A)** and subsurface **(B)** soils. In both panels, the different symbols stand for the different N treatments, i.e., ○ indicates control, △ indicates ammonium addition, ▽ indicates nitrate addition, and □ indicates urea addition. The different colors indicate incubation temperatures, with white used for the 10°C incubation, gray for the 20°C incubation and black for the 30°C incubation.

After the 90-day incubation, the PLFA-derived soil microbial community composition consistently shifted downward along the second PC axis as the temperature increased, but the data within the plots did not clearly assemble in accordance with the applied N form to separate from the data in the control plots (**Figure 3**). Furthermore, ANOVA of the first PCs showed that N treatment had no significant effects on the soil microbial PLFA profiles (*p* > 0.05). The change was accompanied by alterations in the total PLFAs, relative abundance of microbial groups, and the ratios of fungal to bacterial PLFAs (F/B) and of G+ to G- bacterial PLFAs (G+/G-), as indicated by significant correlations between the first two PCs and these microbial properties (*p* < 0.01, **Table 3** and Supplementary Table S2). In particular, PC1 had significant correlations with the total, actinomycetal, G- and fungal PLFAs, F/B, and G+/G- (*p* < 0.05), while PC2 was significantly correlated with the G+, G-, actinomycetal and fungal PLFAs, F/B, and G+/G- (*p* < 0.05, **Table 3** and Supplementary Table S2).

**Table 3:** Results of correlations between the first two PCs and the microbial community properties.

		Total PLFAs	G+	A	G-	F	F/B	G+/G-
PC1	*r*	–0.910^∗∗^	–0.002	–0.747^∗∗^	–0.828^∗∗^	–0.910^∗∗^	–0.827^∗∗^	0.767^∗∗^
	*p*	<0.001	0.987	<0.001	<0.001	<0.001	<0.001	<0.001
	*n*	96	96	96	96	96	96	96
PC2	*r*	0.079	–0.645^∗∗^	0.267^∗∗^	0.464^∗∗^	0.325^∗∗^	0.408^∗∗^	–0.577^∗∗^
	*p*	0.445	<0.001	0.008	<0.001	0.001	<0.001	<0.001
	*n*	96	96	96	96	96	96	96

*PC1 is the first principal component and PC2 is the second principal component extracted by the principal component analysis on the PLFA profiles. G+ stands for Gram-positive bacterial PLFAs, A for actinomycetal PLFAs, G- for Gram-negative bacterial PLFAs, and F for fungal PLFAs. The correlation coefficients *r*, significance level *p* and sample size *n* are shown in the table. The upper ^∗∗^ indicates that the correlation coefficients are significant at the *p* < 0.01 level (2-tailed).*

**FIGURE 4:**
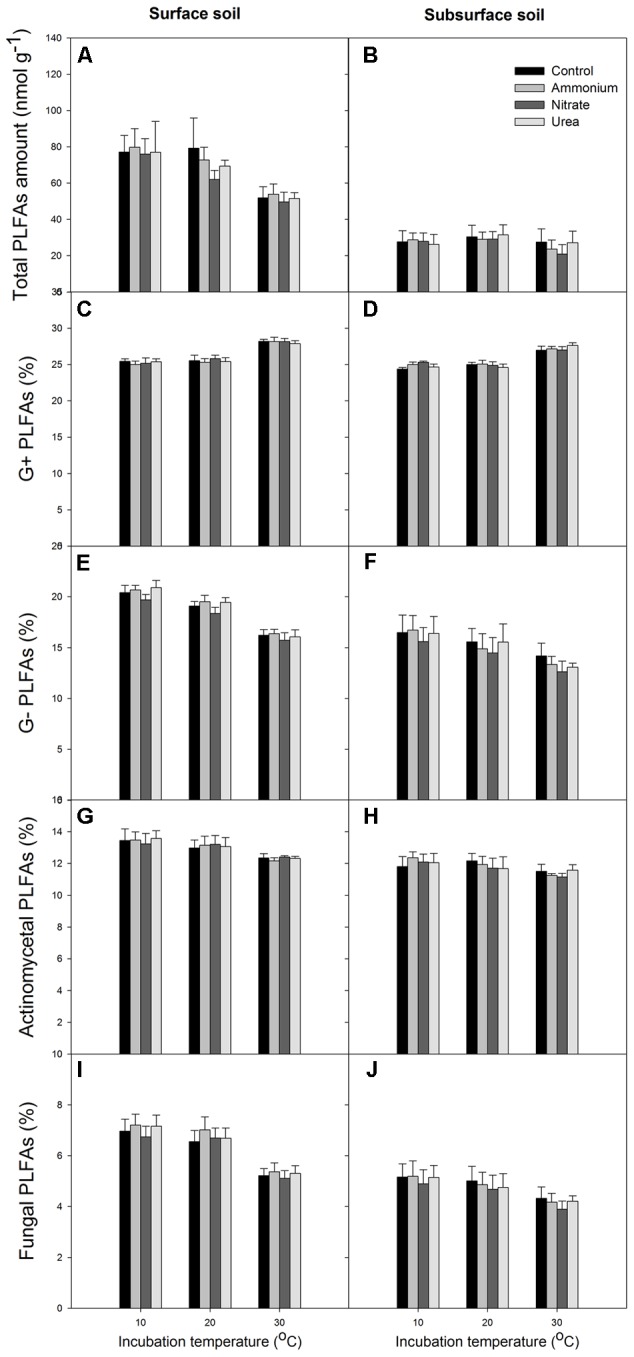
Total amount of PLFAs and relative abundance of the different microbial groups for the surface (left column) and subsurface (right column) soils. **(A,B)** Total PLFAs; **(C,D)** G+ bacterial PLFAs; **(E,F)** G- bacterial PLFAs; **(G,H)** actinomycetal PLFAs; **(I,J)** fungal PLFAs. In each panel, the bars represent the means, and the error bars indicate the standard errors (*n* = 4).

**FIGURE 5:**
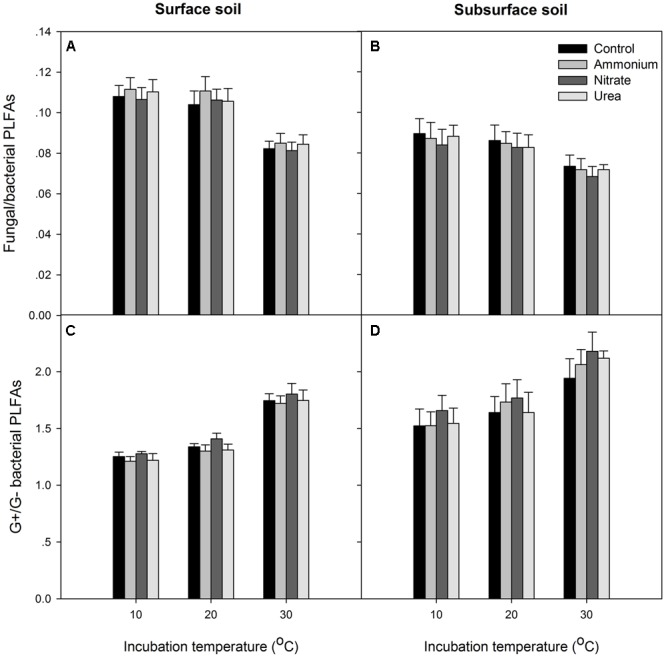
The ratios of fungal to bacterial PLFAs and Gram-positive (G+) to Gram-negative (G–) bacterial PLFAs for the two soils. **(A,B)** The fungal to bacterial ratio; **(C,D)** the Gram-positive to Gram-negative bacterial ratio. In each panel, the bars represent the means, and the error bars indicate the standard errors (*n* = 4).

At the end of the incubation, N addition did not significantly alter the relative abundance of microbial groups in any of the treated soils relative to the control, and N form effect was not significant (*p* > 0.05, **Figures 4, 5**). Temperature significantly affected the total PLFAs as well as the relative abundances and ratios of the microbial groups (**Figures 4, 5**). Briefly, the relative abundance of G+ and the G+/G- PLFA ratio significantly increased with temperature (*p* < 0.05). The total, G- and fungal PLFAs and F/B were significantly lower under the higher temperature than under the lower temperature for the surface soil (*p* < 0.05). Likewise, consistent trends were observed for the subsurface soil, but most of the differences were not significant (*p* > 0.05). Moreover, the subsurface soil had significantly lower microbial biomass, which was indicated by both the fumigation and the PLFA methods (**Table 1** and comparing **Figures 4A,B**), and lower relative abundances of G- and fungal PLFAs (**Figure 4**). This produces lower F/B but higher G+/G- under all of the experimental combinations of temperature and N form (**Figure 5**).

## Discussion

### N Addition Effects on Soil Microbial Respiration

In this study, we found that only urea addition increased soil CO_2_ release in the early stage at 10 and 20°C, while ammonium and nitrate additions did not significantly affect soil CO_2_ emissions in the type of N-rich soils (**Figure 1** and Supplementary Figures S1, S2). The significant increase in soil CO_2_ emissions by urea addition could be derived from the CO_2_ released via hydrolysis of the added urea in the early stage, as urea is an organic material that can be rapidly hydrolyzed after it is applied to soils. When the first peak points were excluded, the effects of N addition were consistently insignificant under all experimental combinations, suggesting that the current N load may not affect soil C emissions. The neutral N effect could be associated with the comparable soil microbial biomass among the treatments (**Figures 4A,B**). This observation is consistent with a previous report in which the addition of 50 kg N ha^-1^ yr^-1^ (corresponding to the N deposition load at the site) and an even greater N load (100 kg N ha^-1^ yr^-1^) did not significantly alter the soil respiration or MBC in the studied forest (Mo et al., 2008), although the plant community diversity and litter decomposition may have been affected (Mo et al., 2006; Lu et al., 2010). Similarly, Deng et al. (2010) reported that a 3-year N addition at a load of 100 kg N ha^-1^ yr^-1^ did not significantly affect soil respiration in mimicked young forest ecosystems where the soils were collected from a nearby subtropical evergreen broadleaved forest, although the above- and belowground biomass was higher than that in the control plots. These results, which combine laboratory soil culture and field investigations in young and mature forests, suggest that the soil microbial communities that live in N-rich soils could have been acclimated to the high N conditions and therefore could be highly resistant to a certain amount of extra N input. However, such microbial resistance likely depends on plant growth conditions and substrate needs (e.g., C and N supplies) (Deng et al., 2010).

Previous studies showed that N deposition could depress soil respiration in some ecosystems (Janssens et al., 2010; Chen et al., 2015), while it could stimulate or not affect soil CO_2_ emissions in other ecosystems (Allison et al., 2008; Zhang et al., 2014). It is likely that the effects of N deposits on soil CO_2_ release are context-specific and depend greatly on the deposition load and resistance/resilience of the ecosystem components (e.g., tolerance of soil microbial communities) (Knorr et al., 2005; Treseder, 2008; Janssens et al., 2010). In spite of the insignificant N effects observed in this study, it is probable that soil CO_2_ emissions will be depressed under high N deposition loads as the N deposition increases in this region (Mo et al., 2008; Liu et al., 2013). Soil microbial communities could respond to atmospheric N deposition non-linearly (Zhou et al., 2012; Gao et al., 2016), and there may be a threshold beneath which N deposition will not significantly affect the composition of the soil microbial groups (He et al., 2016). Once the threshold is exceeded, the soil microbial biomass, community activities and diversity will significantly decline (Zhou et al., 2012). However, this threshold varies across different ecosystems and can encompass 2- to 10-fold of the atmospheric N deposition load in studied ecosystems (Mo et al., 2008; Gao et al., 2016; He et al., 2016).

The addition of N decreased the temperature sensitivity of soil CO_2_ emissions in this study (**Figure 2**), an observation that is consistent with previous studies (Ramirez et al., 2012; Zhang et al., 2014). However, previous studies in the same region suggest that a certain N load threshold (<150 kg N ha^-1^ yr^-1^) could exist, and excess N load over the threshold may reduce the temperature sensitivity of soil respiration in such N-rich forests (Mo et al., 2008), as no significant *Q*_10_ decrease was observed under the 100 kg N ha^-1^ yr^-1^ N addition treatment in either young or mature forest (Mo et al., 2008; Deng et al., 2010). Our results in which the temperature sensitivity of soil microbial respiration was significantly lower with N treatments than with the control treatment probably indicates that soil microbial communities are more sensitive to atmospheric N deposition than are plants. The significant N effect diminished when the soil CO_2_ emissions were divided by the total amount of PLFAs accordingly, indicating an underlying association between *Q*_10_ and soil microbial biomass (Thiessen et al., 2013). Alternatively, this *Q*_10_ reduction could be due to the inhibition of specific microbial species, the adjustment of microbial physiology or shifts in substrate preference under N addition (Treseder, 2008; Allison et al., 2010; Ramirez et al., 2012). Recalcitrant organic matter (OM) is mainly decomposed to obtain N for microbial growth and maintenance by specific microbial groups (Craine et al., 2007; Ramirez et al., 2012). After the easily utilizable N-containing materials were applied, the N inhibition effect and the shift in substrate preference resulted in less decomposition of recalcitrant OM (Carreiro et al., 2000; Treseder, 2008; Du et al., 2014), consequently decreasing the *Q*_10_ due to the lower temperature dependency of labile OM than recalcitrant OM (Conant et al., 2008; Xu et al., 2010). This is also evidenced by the observation that the *Q*_10_ exhibited the greatest decrease with the addition of urea in the early stage because urea is an easily utilizable organic substrate for soil microorganisms.

### Effects of N Form

In contrast to our expectations, we observed that the N effect was independent of N chemistry, as the N type did not cause significantly different soil microbial PLFA compositions (**Figures 3**–**5**). The chemistry of the N source added to the soil could affect the microbial activities and respiration (Liu and Greaver, 2010; Du et al., 2014) due to different N substrate preferences (Bardgett et al., 2003), whereas consistent N effects for ammonium, nitrate and urea have also been observed (Ramirez et al., 2010a). It is difficult to completely explain the discrepancy on account of the many underlying factors. In the studied forest, however, the independence could be partly attributed to the continuously high N deposition load and the N-saturated soil conditions (Fang et al., 2008; Chen et al., 2015). Microorganisms that live in N-excess soils could be acclimated to the N-enriched environment. Therefore, exogenous N input at the current deposition rate may not have induced any change in the PLFA-derived soil microbial community composition, regardless of the application of ammonium, nitrate, or urea. However, this result should be interpreted with caution, as the comparable PLFA profiles do not necessarily mean that N form did not significantly affect the soil microbial community composition at other organization levels, e.g., the phylogenetic level. In actuality, the PLFA method is not sensitive enough to detect phylogenetic shifts in soil microbial communities, although it is useful for detecting phenotypic changes (Frostegård et al., 2011). Moreover, our observations do not deny the possibility that a different combination of N load and duration could result in microbial community shifts because soil microbial communities depend greatly on N treatment load and duration (Treseder, 2008; Gao et al., 2016; Wang et al., 2017), consequently changing C cycling (Liu and Greaver, 2010).

Ammonium is the substrate for microbial nitrification, an N transformation process that could potentially enhance the within-soil CO_2_ uptake (Fleischer and Bouse, 2008). In contrast, nitrate may retard the nitrification process due to it being the product of nitrification, and could therefore increase soil CO_2_ emissions in accordance with the Le Chatelier principle (Fleischer and Bouse, 2008; Fleischer, 2012). In our study, several PLFAs showed consistent trends in response to N additions, e.g., compared with the control and ammonium addition treatments, nitrate addition reduced the relative abundance of 16:1w7c and 18:1w7c. Such decreases could reduce nitrification rates, as previous studies have suggested that these two PLFAs are dominant fatty acids in N-oxidizing bacteria such as *Nitrosomonas* and *Nitrobacter* (Petersen et al., 2004; Börjesson et al., 2011). Consequently, soil CO_2_ emissions may increase due to the reduced within-soil CO_2_ uptake (Fleischer and Bouse, 2008; Fleischer, 2012), which is partially supported by the observation that nitrate additions consistently induced higher soil CO_2_ emissions. This result implies that the decrease in the ammonium/nitrate ratio of atmospheric N deposition (Liu et al., 2013) will tend to cause higher soil CO_2_ emissions, which increases risk of further accelerating warming by inducing greater positive feedback.

Moreover, although the potential mechanisms remain poorly understood (Sinsabaugh et al., 2005; Cusack, 2013), the ammonium/nitrate ratio positively affects the activities of oxidative enzymes (Cusack, 2013). Such a positive correlation could be due to the inhibition of oxidative enzyme activities by nitrate addition (Saiya-Cork et al., 2002; Sinsabaugh et al., 2005); this phenomenon is often associated with losses of soil microbial biomass (such as white rot basidiomycetes) and reduced microbial investment to produce oxidative enzymes (Sinsabaugh et al., 2005; Sinsabaugh, 2010; Cusack, 2013). Furthermore, reductions in soil oxidative enzyme activity combined with a general increase in the activity of hydrolytic enzymes (e.g., α-glucosidase and cellobiohydrolase) indicate a more rapid turnover and a diminishing stock of labile OM but a slower decomposition of recalcitrant organic compounds (Saiya-Cork et al., 2002; Sinsabaugh et al., 2005). Even though SOM fractions were not investigated in this study, the decreased ammonium/nitrate ratio could be beneficial to the accumulation of recalcitrant OM. This phenomenon further increases the uncertainties of predicting the soil CO_2_ emissions under the changing N deposition composition in the future.

### Comparisons of N Effects between Soil Layers

Within the study period, the CO_2_ emissions from the surface soil responded more to higher temperatures than the subsurface soil (**Figure 2**). This is opposite from previous observations in two boreal upland forests in which the subsoil had higher *Q*_10_ values than did the surface soil (Karhu et al., 2010). In the present study, this *Q*_10_ difference was mainly attributed to the lower soil microbial biomass in the subsurface soils than in the surface soils (**Table 1** and **Figures 4A,B**) because the temperature sensitivity of decomposition greatly depends on soil microbial biomass (Thiessen et al., 2013). This explanation is well supported by our observation that the difference between the surface and subsurface soils became not significant after dividing the CO_2_ emission by the corresponding total PLFA amounts.

Moreover, the effects of N on the soil microbial communities were consistent between the two layers of soil that exhibited different soil properties. This result does not follow our expectations, albeit soil properties (especially C and N availability) have been suggested to greatly affect the microbial responses to environmental changes (Karhu et al., 2010; Eberwein et al., 2015). This dependence, however, remains inconclusive according to the existing knowledge. Previous studies have also reported consistent N effects on microbial processes and community dynamics across contrasting soils (Ramirez et al., 2010b, 2012), which was also observed in this study. These results suggest that N effects on soil microbial properties could be independent of soil properties in several ecosystems.

Atmospheric N deposition has been reported to decrease *Q*_10_ in diverse ecosystems (Mo et al., 2008; Ramirez et al., 2012; Zhang et al., 2014), while positive effects have also been observed (Deng et al., 2010). Such diverse observations among studies are likely due to the relatively low N fertilization rates that occur under the background N deposition and environmental conditions. For example, Mo et al. (2008) reported that low and medium levels of N addition (up to 100 kg N ha^-1^ yr^-1^) did not change the temperature sensitivity of soil respiration, but the addition of 150 kg N ha^-1^ yr^-1^ significantly decreased the *Q*_10_ in the studied forest where atmospheric N deposition was approximately 50 kg N ha^-1^ yr^-1^ (Fang et al., 2008). These observations suggest that N deposition could exert consistent effects on microbial activity and community PLFAs across ecosystems, but the consistency may depend greatly on N deposition load.

## Conclusion

Exogenous N additions similar to the current N deposition load did not significantly affect soil CO_2_ emissions or microbial community PLFAs in the two layers of soil collected from a subtropical forest in southern China. The exception was for urea, which stimulated soil CO_2_ emissions in the early stage. This result implies that soil microbial community PLFAs and activity are resistant to the current deposition loads and the changing composition. However, the addition of N decreased the temperature sensitivity of soil CO_2_ emissions, indicating that atmospheric N deposition has a negative effect on the microbial response to warming. This further highlights the need to clarify the interactive effects of global changes on ecological processes and functions by conducting multiple-factor experiments. Moreover, similar N effects were observed for the soils that exhibited different soil properties, indicating that there may be a consistent microbial resistance to current N deposition loads and composition changes across those N-rich soils.

## Author Contributions

HW and XC carried out the experiment, analyzed the data and wrote the draft manuscript. WS and HW conceived the study. JH and JZ helped with field sampling and laboratory analyses. All authors contributed to manuscript writing and revision.

## Conflict of Interest Statement

The authors declare that the research was conducted in the absence of any commercial or financial relationships that could be construed as a potential conflict of interest.
