# Supplementary material for: Exogenous Nitrogen Addition Reduced the Temperature Sensitivity of Microbial Respiration without Altering the Microbial Community Composition
Source: Front Microbiol. 2017 Dec 1;8:2382. doi: 10.3389/fmicb.2017.02382 (PMC5770617; doi:10.3389/fmicb.2017.02382)
Supplement: Supplementary file 1 [file Presentation_1.PDF]

Supplementary Materials

Exogenous nitrogen addition reduced the temperature sensitivity of microbial respiration without altering the microbial community composition

Running title: N effects on microbial communities

Author: Hui Wei[1,2,3,†], Xiaomei Chen[4,†], Jinhong He[5,6], Jiaen Zhang[1,2,3], Weijun Shen[5,*]

[1] Department of Ecology, College of Natural Resources and Environment, South China Agricultural University, Guangzhou, China

[2] Key Laboratory of Agro-Environment in the Tropics, Ministry of Agriculture, Guangzhou, China

[3] Guangdong Engineering Research Center for Modern Eco-agriculture and Circular Agriculture, Guangzhou, China

[4] School of Geographical Sciences, Guangzhou University, Guangzhou, China

[5] Guangdong Provincial Key Laboratory of Applied Botany, South China Botanical Garden, Chinese Academy of Sciences, Guangzhou, China

[6] University of Chinese Academy of Sciences, Beijing, China

† These authors contributed equally to this work.

* Correspondence: Dr. Weijun Shen

Email: shenweij@scbg.ac.cn
Tel.: +86 20 3725 2950
Fax: +86 20 3725 2950

Table S1 Summary of analysis of covariance (ACOVA) on the soil CO_2 emission in each of the two soils. In ANCOVA, the accumulated soil CO_2 emission was used as the dependent variable, with N form being a fixed factor and incubation temperature (T) being a covariate. Important model parameters including sum of squares (*SS*), degree of freedom (*df*), *F* and *p* value are also given in the table.

	Surface soil				Sub-surface soil			
	SS	*df*	*F*	*p*	*SS*	*df*	*F*	*p*
N form	921.45	3	0.67	0.578	377.51	3	0.30	0.826
T	55783.20	1	121.04	<0.001	18509.98	1	43.86	<0.001
Interaction	767.06	3	0.56	0.648	346.58	3	115.53	0.844

Table S2 Loading scores of the first two principal components (PC) derived from a principal component analysis on the PLFAs profile. In the table, PC1 is the first principal component and PC2 is the second principal component.

PLFA category	PLFAs	PC1	PC2
G+ PLFAs	13:0 anteiso	0.020	0.034
	14:0 iso	0.008	-0.048
	14:0 anteiso	0.002	-0.029
	15:0 iso	-0.032	0.035
	15:0 anteiso	0.028	-0.011
	16:0 iso	-0.035	-0.102
	17:0 iso	0.061	-0.025
	17:0 anteiso	0.063	0.033
	18:0 iso	0.066	0.013
	19:0 iso	0.065	-0.024
G- PLFAs	17:0 cyclo	0.010	0.074
	19:0 cyclo w8c	-0.067	0.003
	14:1 w5c	-0.005	-0.025
	15:1 w5c	-0.028	0.018
	16:1 w7c	-0.014	0.119
	18:1 w7c	-0.028	0.108
	16:0 2OH	0.033	0.029
Actinomicetal PLFAs	16:0 10-methyl	-0.065	0.027
	17:0 10-methyl	-0.057	-0.042
	18:0 10-methyl	0.048	0.049
Fungal PLFAs	18:2 w6c	-0.038	0.079
	18:1 w9c	-0.065	0.031
Undefined bacterial PLFAs	16:1 w5c	-0.053	0.020
	14:00	0.037	-0.046
	15:00	0.004	-0.118
	17:00	-0.037	-0.077
	18:00	0.007	-0.090
Undefined microbial PLFAs	16:00	-0.049	-0.086
	15:4 w3c	-0.008	0.083
	15:1 w8c	0.003	0.019
	16:4 w3c	0.034	-0.083
	16:3 w6c	0.068	0.005
	16:2 DMA	-0.001	-0.073
	16:1 w9c DMA	-0.001	-0.001
	17:1 iso w9c	0.068	0.017
	17:1 w8c	-0.025	0.022
	17:1 w7c 10-methyl	0.065	0.025
	18:3 w3c	0.049	0.008
	18:1 w7c 10-methyl	0.025	0.083
	19:1 w7c 10-methyl	0.036	-0.038

FIGURE CAPTION

Figure S1. Instantaneous CO_2 efflux rate under the different experimental treatments for the surface soil within the investigation period. The signals represent the means, and the error bars are the standard errors (n=4).

Figure S2. Instantaneous CO_2 efflux rate under the different experimental treatments for the sub-surface soil within the investigation period. The signals represent the means, and the error bars are the standard errors (n=4).

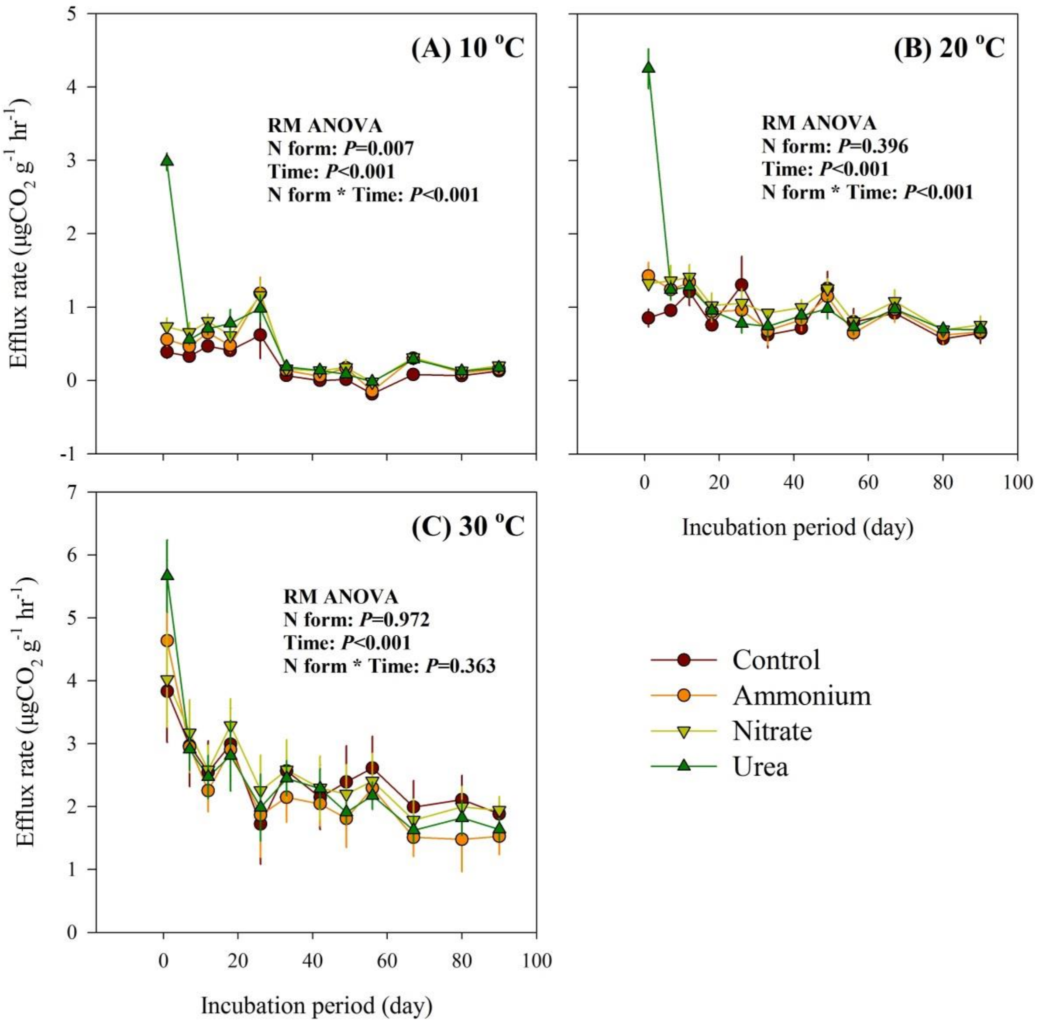
(A) 10 °C
RM ANOVA
N form: P=0.007
Time: P<0.001
N form * Time: P<0.001
(B) 20 °C
RM ANOVA
N form: P=0.396
Time: P<0.001
N form * Time: P<0.001
(C) 30 °C
RM ANOVA
N form: P=0.972
Time: P<0.001
N form * Time: P=0.363
Efflux rate (μgCO2 g-1 hr-1)
Incubation period (day)
Control
Ammonium
Nitrate
Urea

Figure S1

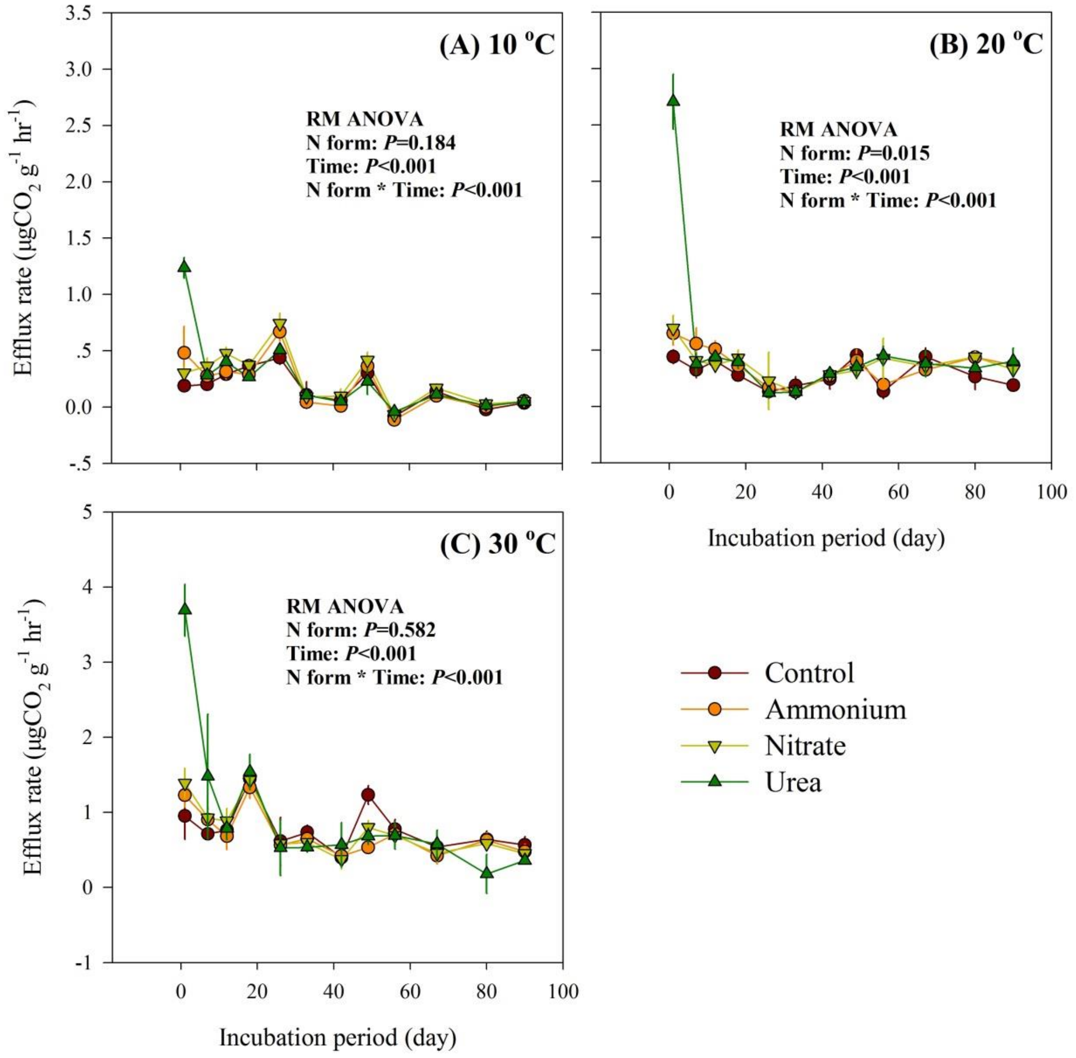
(A) 10 °C
RM ANOVA
N form: P=0.184
Time: P<0.001
N form * Time: P<0.001
(B) 20 °C
RM ANOVA
N form: P=0.015
Time: P<0.001
N form * Time: P<0.001
(C) 30 °C
RM ANOVA
N form: P=0.582
Time: P<0.001
N form * Time: P<0.001
Efflux rate (μgCO2 g-1 hr-1)
Incubation period (day)
Control
Ammonium
Nitrate
Urea

Figure S2
